# A Neuronal Transcriptome Response Involving Stress Pathways is Buffered by Neuronal microRNAs

**DOI:** 10.3389/fnins.2012.00156

**Published:** 2012-10-30

**Authors:** Sergei A. Manakov, Andrew Morton, Anton J. Enright, Seth G. N. Grant

**Affiliations:** ^1^Genes to Cognition Programme, Wellcome Trust Sanger InstituteCambridge, UK; ^2^RNA Genomics Lab, European Molecular Biology Laboratory-European Bioinformatics InstituteCambridge, UK; ^3^Centre for Integrative Physiology, University of EdinburghEdinburgh, UK

**Keywords:** miRNA, transcriptome, neuronal stress, buffering, primary neuronal culture

## Abstract

A single microRNA (miRNA) can inhibit a large number of mRNA transcripts. This widespread regulatory function has been experimentally demonstrated for a number of miRNAs. However, even when a multitude of targets is confirmed, function of a miRNA is frequently interpreted through a prism of a handful arbitrarily selected “interesting” targets. In this work we first show that hundreds of transcripts with target sites for two miRNAs expressed endogenously in neurons, miR-124 and miR-434-3p, are coordinately upregulated in a variety of neuronal stresses. This creates a landscape where these two miRNAs can exert their widespread inhibitory potential on stress-induced transcripts. Next, we experimentally demonstrate that overexpression of these two miRNAs indeed significantly inhibits expression of hundreds of stress-induced transcripts, thus confirming that these transcripts are enriched in true targets of examined miRNAs. A number of miRNAs were previously shown to have important roles in the regulation of stress responses, and our results suggest that these roles should be understood in light of a wide spread activation of miRNA targets during stresses. Importantly, a popular cationic lipid transfection reagent triggers such induction of miRNA targets. Therefore, when a transfection paradigm is employed to study miRNA function, the results of such studies should be interpreted with consideration for the inadvertent induction of miRNA targets.

## Introduction

Binding to a short specific sequence motif (∼7 nt long) within 3′untranslated regions (UTR) of mRNAs underlies recognition of targets by microRNA (miRNAs; Lewis et al., 2003, 2005). The presence of these motifs, called seed matching sites or a target sites, in many mRNAs allows miRNA to have many targets. The scale of targeting repertoires of individual repertoires was first predicted computationally (Enright et al., 2003; Lewis et al., 2003; Stark et al., 2003) and then demonstrated experimentally (Lim et al., 2005; Giraldez et al., 2006). However, despite having multiple targets, the function of a miRNA is frequently interpreted by considering only one or few, sometimes arbitrary selected, targets (Makeyev et al., 2007; Visvanathan et al., 2007; Rybak et al., 2008). Even though focusing on one or two targets gives detailed insights into specific functional aspects of a miRNA in question, all targets should be considered in an unbiased fashion to achieve a proper understanding of the role of a miRNA.

Their multiple targets make miRNAs perfectly suited to canalize transcriptome changes during development, as well as to maintain the stability of differentiated states by buffering transcriptional perturbations (Hornstein and Shomron, 2006). Deviations from the normal homeostatic state of the transcriptome occur during stresses, and indeed miRNAs were shown to impart robustness to stress responses. For example, miR-7 is necessary for eye development under temperature fluctuations (Li et al., 2009), miR-14 for survival during salt stress (Xu et al., 2003), miR-8 for adaptations to osmotic fluctuation (Flynt et al., 2009). More examples of miRNAs with confirmed roles in stress are reviewed elsewhere (Leung and Sharp, 2010). However, in the extensive body of work on miRNAs in stresses, research has typically focused on a selected few targets, and the relationship between the complete repertoires of targets and changes in the transcriptome during stress is still largely not understood.

The neuronal transcriptome is a relevant and important context in which to explore miRNA function. The evolutionary origin of neurons was linked to the emergence of a neuron specific miRNA, miR-124 (Christodoulou et al., 2010), and this and other neuronal miRNAs are critically important for neuronal function (Rajasethupathy et al., 2009; Schratt, 2009). Therefore, we decided to study the link between miRNAs and transcriptome wide changes in gene expression in primary neuronal cultures, a popular system for studying neuronal biology (Nelson, 1975; Dichter, 1978). By transfecting miRNA inhibitors and mimics, we interfered with specific miRNAs in primary cultures of cortical neurons and then identified the sets of regulated mRNAs using transcriptome profiling. This experimental method of identifying miRNA targets has been previously validated and published (Lim et al., 2005; Giraldez et al., 2006). With this approach we found that hundreds of transcripts induced by stresses of neurons and the brain are in fact direct targets of miRNAs. Targets of neuronal miRNAs, miR-124 and miR-434-3p, are enriched in genes that are activated by stresses, and, importantly, this enrichment is higher than is the case for non-neuronal miRNAs. Hence these two neuronal miRNAs have a capacity to buffer massive changes in the neuronal transcriptome that occur during stresses.

In addition, we report that the widely used neuronal transfection procedure promotes a major stress response. Treatment of primary neurons with blank cationic lipid transfection reagent alone (Materials and Methods) induced expression of stress-related genes enriched in miR-124 target sites in their transcript 3′UTRs. We experimentally confirmed that a significant proportion of these transcripts are indeed targeted by miRNAs, with targets of two neuronal miRNAs, miR-124 and miR-434-3p being the most enriched. Therefore our study raises a note of caution for those studying miRNAs, as we found that cationic lipid transfection, a commonplace experimental procedure, inadvertently induces expression of miRNA targets.

## Materials and Methods

### Primary neuronal cultures

All mice were treated in accordance with the UK Animals (Scientific Procedures) Act of 1986, and all procedures were approved through the UK Home Office Inspectorate. Pregnant C57BL/6 c/c mice at 17.5 days gestation were sacrificed and the forebrains of decapitated embryos were dissected. Forebrains were shredded and transferred to papain (10 units/mL, Worthington) for 20 min at 37°C. Cells were dispersed by trituration in DMEM (Invitrogen) and centrifuged at 400 *G* for 3.5 min. The pellet was resuspended in Neurobasal (Invitrogen) supplemented with B-27 (Invitrogen) and 0.5 mM l-Gln (Invitrogen). The suspension was centrifuged at 400 *G* for 3.5 min. The final pellet was resuspended in the supplemented Neurobasal. The cells were plated onto poly-d-lysine (Sigma) and laminin (Invitrogen) coated plastic substrates (Corning) at a density of ∼1,300 cells/mm^2^. The cells were cultured in the supplemented Neurobasal in a humidified incubator at 37°C and 5% CO_2_.

### Transfections

Transfections were performed using DharmaFECT 3 transfection reagent (Dharmacon) according to the manufacturer’s protocol as previously described (MacLaren et al., 2011). At 4–6 days after plating, the cultures were transfected with mimics and inhibitors of mouse miRNAs (Qiagen) or the mimic of cel-miR-67 (Dharmacon). All transfections were performed in three or four biological replicates. After ∼48 h post-transfection, the total RNA was extracted from the cultures.

### KCl treatment

At 6 days after plating, KCl was added to the culture media to a final concentration of 15 mM. After ∼48 h, total RNA was extracted from cultures treated with KCl and matched untreated cultures (eight biological replicates per condition). Gene expression was profiled with Illumina microarrays (see below). Differentially expressed transcripts where defined as those corresponding to probes with multiple testing adjusted *P* < 0.01. *P*-values were adjusted with Benjamini and Hochberg (1995) procedure as implemented via limma package functions (Smyth, 2004). This procedure was also followed in all other cases when it is stated that *P*-value adjustment was performed (see below).

### RNA extraction and microarray profiling

At ∼48 h post-transfection or post-treatment with 15 mM KCl, the culture media was removed and the cells were lysed with QIAzol (Qiagen). Total RNA was extracted with miRNeasy Mini Kit (Qiagen) according to the manufacturer’s protocol. Profiling of mRNA abundances was performed using an Illumina Sentrix BeadChip Array Mouse-WG6 v2 microarray platform according to the Illumina protocol.

### Analysis of microarray data

Illumina arrays were scanned using a BeadArray reader (Illumina) and raw probe intensities were exported with GenomeStudio software (Illumina) according to the manufacturer’s protocols. The raw data was deposited in ArrayExpresses (Parkinson et al., 2009; accession number E-MTAB-68). Analysis of the array data was performed in the R environment (R. Team, 2008) with Bioconductor packages (Gentleman et al., 2004). The output of GenomeStudio was imported into R using lumi package functions (Du et al., 2008). Only probes with lumi detection call *P* < 0.01 were considered for further analysis. The Illumina array data were transformed with VST and normalized with RSN as implemented via the lumi package. Analysis of differential gene expression was performed using limma package functions (Smyth, 2004).

Microarray data from KCl treatment and transfection experiments is deposited in ArrayExpress^1^ under E-MTAB-686 accession.

Raw Affymetrix microarray mRNA profiling data for the kainate injection experiment (Akahoshi et al., 2007) was downloaded from GEO (Barrett et al., 2011; accession number GSE6388). The raw data was imported into R environment, transformed and normalized with RMA as implemented via the affy package (Gautier et al., 2004). Analysis of differential gene expression was performed using limma package functions (Smyth, 2004). Probes with differential expression *P* < 0.05 were considered to be differentially expressed.

#### Mapping Illumina microarray probes to transcript and gene identifiers

Illumina microarray probe sequences were taken from the Illumina mRNA array annotation file MouseWG-6_V2_0_R2_11278593_A (BGX file) available from the Illumina website^2^. Illumina mRNA array probes were aligned to the complete set of the full-length Ensembl v56 mouse transcripts (Flicek et al., 2008; Hubbard et al., 2009) with SSAHA2 (Ning et al., 2001). All categories of Enesmbl transcripts, were retrieved using the Ensembl Perl API, which enabled access to Core, Vega, and OtherFeatures mouse transcripts (Flicek et al., 2008; Hubbard et al., 2009). A transcript from the highest scoring SSAHA2 alignment was chosen for each probe (at least 30 perfect consecutive matches were required). If more than one alignment had an equally high score, manually curated Vega transcripts were preferred. In order to resolve ambiguity of multiple transcripts from the same source aligning equally well, the “biotype” annotation was considered (protein coding transcripts were preferred to pseudogenes, and nonsense-mediated decay had the lowest preference). If the ambiguity was still not resolved, transcripts with the longest 3′UTR or cDNA were selected. When probes did not align to any of the Ensembl transcripts, mapping of the probes to RefSeq 38 (Pruitt et al., 2009) transcript identifiers was taken from the Illumina BGX files. After probes were uniquely mapped to the best transcript identifiers (either from Ensembl or from the Illumina BGX files), the corresponding GeneBank gene identifiers (referred to as Entrez gene IDs) were matched to the probes. For probes mapped to Ensembl or Vega transcripts, the Entrez gene IDs were retrieved using Ensembl API, and for probes mapped to RefSeq IDs via the BGX annotation files, the Entrez gene IDs were taken from the BGX file. The adjusted *P*-values of differential expression were used for selection of the best probe per Entrez gene ID (i.e., a probe with the most significant adjusted *P*-value was preferred).

#### Mapping Affymetrix microarray probes to transcript and gene identifiers

Bioconductor annotation library “mgu74bv2.db” (Gentleman et al., 2004) was used in analysis of the Affymetrix data (Akahoshi et al., 2007). These libraries provided mapping of microarray probes to RefSeq transcript and Entrez gene identifiers. For RefSeq transcript IDs, 3′UTR sequences were obtained from Ensembl, using Ensembl API (Flicek et al., 2008; Hubbard et al., 2009). The length of 3′UTRs was used to resolve the ambiguity in mapping of probes to RefSeq transcript IDs: when the probes were mapped to more than one RefSeq transcript ID, the ID corresponding to the transcript with the longest 3′UTR was selected. In the Bioconductor annotation files, each RefSeq transcript ID corresponded to one Entrez gene ID. The adjusted *P*-values of differential expression were used for selection of the best probe per Entrez gene ID (i.e., a probe with the most significant adjusted *P*-value was preferred).

#### Obtaining 3′UTR sequences of transcripts

The 3′UTR sequence for the transcripts mapped to each of the microarray platforms were obtained from Ensembl v56 with the Ensembl API (Flicek et al., 2008; Hubbard et al., 2009). For Sylamer analysis regions of low complexity and repetitive (redundant) sequences were removed as previously described (van Dongen et al., 2008). The low complexity and redundant sequences were masked out (Thomas-Chollier et al., 2008).

### Sylamer analysis

The sequences of all mature mouse miRNAs were downloaded directly from miRBase Release 14 (Griffiths-Jones et al., 2008). For each miRNA, two sequences complementary to the seed region (i.e., the seed matching sites) were derived: the sequence complementary to bases 2–8 from the 5′-end of the miRNA [7(2)-type seed matching site], and to bases 1–7 with an A opposite to position 1 [7(1A)-type; Bartel, 2009] producing 876 distinct seed matching sites in total. Three seed matching sites, CAAUAAA, UAUUUAU, and UCAAUAA that are similar to the AAUAAA, a polyadenylation signal in 3′UTRs (Connelly and Manley, 1988) had to be excluded from the analyzes, as biases in distribution of these words could not be attributed to miRNA-mediated effects.

Sylamer tests for nucleotide word occurrence biases in a sorted list of sequences using hypergeometric test and is suitable to profile biases in distribution of miRNA seed matching sites in the list of 3′UTRs (van Dongen et al., 2008). Assessment of the enrichment of a particular nucleotide word of a given length is done by calculation of a hypergeometric enrichment *P*-value of that word in samples of sequences (or bins) from the list. Sampling of sequences from the ordered list was done from the most downregulated to the most upregulated and the size of a leading bin was iteratively incremented (i.e., at each step the leading bin includes all previously sampled sequences plus a certain number of new sequences). At each step, Sylamer calculates the enrichment *P*-value of a particular nucleotide word by comparing its occurrence in the leading bin to its occurrence in all sequences of the list. Transcripts without a 3′UTR sequence were excluded from the test.

Sequences were sorted by *t*-statistic of corresponding microarray probes. In each test, the distribution of 876 seed matching sites of length seven bases corresponding to mouse mature miRNAs (see above) was assessed. The sample size (bin size) of selected sequences was incremented by 100 at each step. The level of Markov-correction was set to four (van Dongen et al., 2008).

Putative direct targets of miRNAs were defined as previously described (van Dongen et al., 2008). Briefly, first, a *P*-value cutoff for differential expression was selected. We selected the cutoff to be equal to 0.05, because this adjusted *P*-value for differential expression roughly coincided with the peak of Sylamer enrichment of seed matching sites for transfected miRNAs in a majority of experiments (Figure 3). Second, transcripts downregulated (adjusted *P* < 0.05) in contrast between transfections of miRNA mimics and miRNA inhibitors (for all mouse miRNAs) or mock transfection (for cel-miR-67) were selected. Finally, putative direct targets of a transfected miRNA were defined as a sub-selection of these transcripts that contained one or more seed matching sites for transfected miRNAs.

## Results

### Induction of stress response genes by the transfection reagent

We proceeded to use a standard transfection protocol, utilized in thousands of studies, where a mixture of a cationic lipid and nucleic acid is applied to the cultured neurons. In the course of analyzing mRNA profiles from multiple different miRNA inhibitors and mimics, we were initially surprised by a set of mRNAs that unexpectedly changed in all experiments. In analyzing this phenomenon, we tested the mRNA profile of neurons treated with cationic lipid alone and compared this with untreated neurons and unexpectedly found the transfection reagent alone induced a major change in the transcriptome.

**Figure 1:**
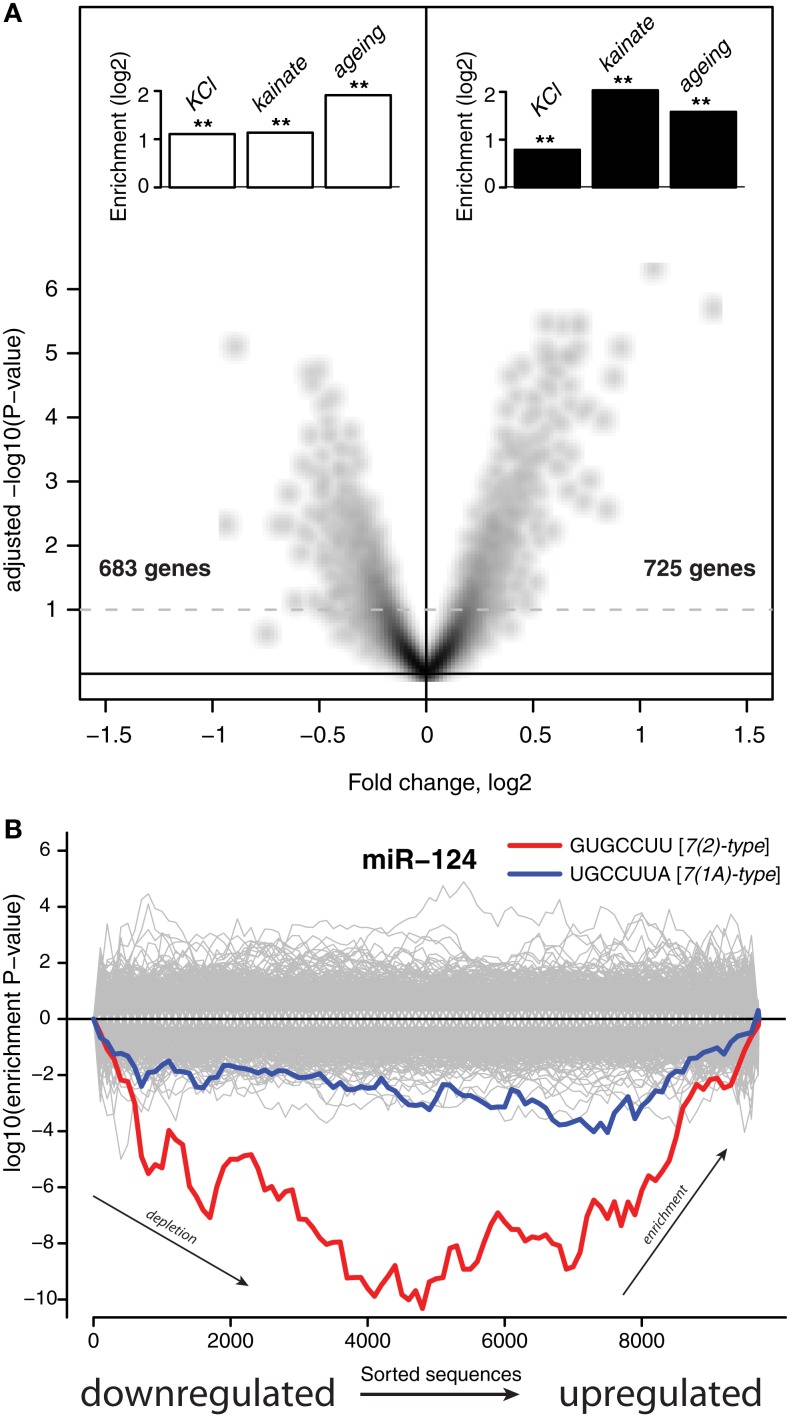
**Stress induces expression of genes subject to miRNA-mediated regulation. (A)** The *x*-axis shows differential expression (log_2_) between mock transfected and untransfected cultures, the *y*-axis shows the adjusted *P*-value (**−**log_10_) for differential expression. Each point on the plot uniquely represents one gene, genes above the dashed line are differentially expressed with multiple testing adjusted *P* < 0.1. The insets show enrichment (log_2_) of genes either downregulated (white bars) or induced (black bars) by stressful treatments. Genes differentially expressed upon KCl treatment where identified by Illumina microarrays (Materials and Methods). Genes differentially expressed upon kainite treatment where derived from published microarray profiling data (Akahoshi et al., 2007; Materials and Methods). Mouse homologs (from HomoloGene; Sayers et al., 2010) of human genes that were previously reported as differentially expressed upon aging of the human brain comprised the aging sets. Double asterisks indicate hypergeometric *P* **<** 0.001 (Materials and Methods). The set of 10,849 genes detected by Illumina microarrays (Materials and Methods) in mock transfected and untransfected cultures was used as the universe for the hypergeometric tests. **(B)** The *x*-axis represents 3′UTRs corresponding to expressed genes sorted from most downregulated to most upregulated in comparison of mock transfected vs. untransfected cultures. *P*-values are calculated using the Sylamer method (van Dongen et al., 2008). Positive values on the *y*-axes represent nucleotide word enrichment [+|log_10_(*P*-value)|] and negative values represent depletion [−|log_10_(*P*-value)|]. The red and blue lines show enrichment profiles of *7(2) or 7(1A)-type seed matching sites* (Bartel, 2009) for miR-124, the gray lines – for other miRNAs (Materials and Methods).

Figure 1A shows that ∼48 h after addition of the transfection reagent there was a reduction in expression of 683 mRNAs (6.3% of transcriptome) and an upregulation of 725 mRNAs (6.7% of transcriptome). The lists of genes encoding these mRNAs, together with fold changes and *P*-values of differential expression, are supplied in Data Sheet 2 in Supplementary Material. Inspection of these lists revealed many important regulatory genes and KEGG pathway analysis (Kanehisa et al., 2000) showed significant induction in the p53 pathway, P450 related pathway and reduction in lipid biosynthesis and transport. Thus, the treatment with a cationic lipid transfection reagent triggers a major response in the transcriptome involving stress and metabolic pathways.

### Other treatments also induced the response

We next asked if this transcriptome response was relevant to neuronal physiology or simply an idiosyncratic effect of the transfection reagent. To address this, we compared these transfection-regulated mRNAs with transcriptome data from neurons that had been subjected to other types of stressful treatments. First, we analyzed published microarray transcriptome profiling of hippocampi in which glutamate receptors were abnormally activated through injection of kainate (Akahoshi et al., 2007). We identified sets of transcripts with expression either down or upregulated by this treatment (Materials and Methods). Transcripts that were downregulated upon injection of kainate had a significant intersection with transcripts downregulated by the transfection reagent treatment of the neuronal cultures (*P* < 4.62 × 10^−13^, Figure 1A), while transcripts upregulated by the kainate intersected significantly with transcripts upregulated by the transfection reagent (*P* < 3.72 × 10^−39^, Figure 1A). Next, we analyzed lists of genes that were previously reported to be either induced or reduced in expression in the human brain upon aging (Lu et al., 2004). Mouse homologs of human genes were obtained from HomoloGene (Sayers et al., 2010) and their transcripts were compared to transcripts differentially expressed upon the transfection reagent treatment. Again, transcriptome changes observed in the aging brain were similar to those observed upon the transfection treatment (*P* < 1.36 × 10^−09^ for intersection of downregulated transcripts and *P* < 7.73 × 10^−08^ for upregulated transcripts, Figure 1A). Finally, we conducted an experiment were we chronically depolarized primary neuronal cultures by treatment with KCl (Materials and Methods). We identified genes that were either down or upregulated through transcriptome profiling with microarrays (Materials and Methods). As with the published accounts of transcriptome changes in the brain upon kainate injection and aging, KCl treatment of the cultures induced changes in the transcriptome overlapped significantly with those observed upon the cationic lipid transfection reagent (*P* < 4.60 × 10^−39^ for downregulated transcripts and *P* < 8.75 × 10^−18^ for upregulated transcripts, Figure 1A). All the gene lists, and, when available, fold changes and *P*-values of differential expression, are supplied in Data Sheet 2 in Supplementary Material. These observations suggest significant similarity between sets of genes activated in response to different stresses. Therefore, for convenience we refer to the response of neurons to different stresses as Neuronal Challenge Response (NCR).

### The response involves induction of transcripts subject to miRNA-mediated regulation

**Figure 2:**
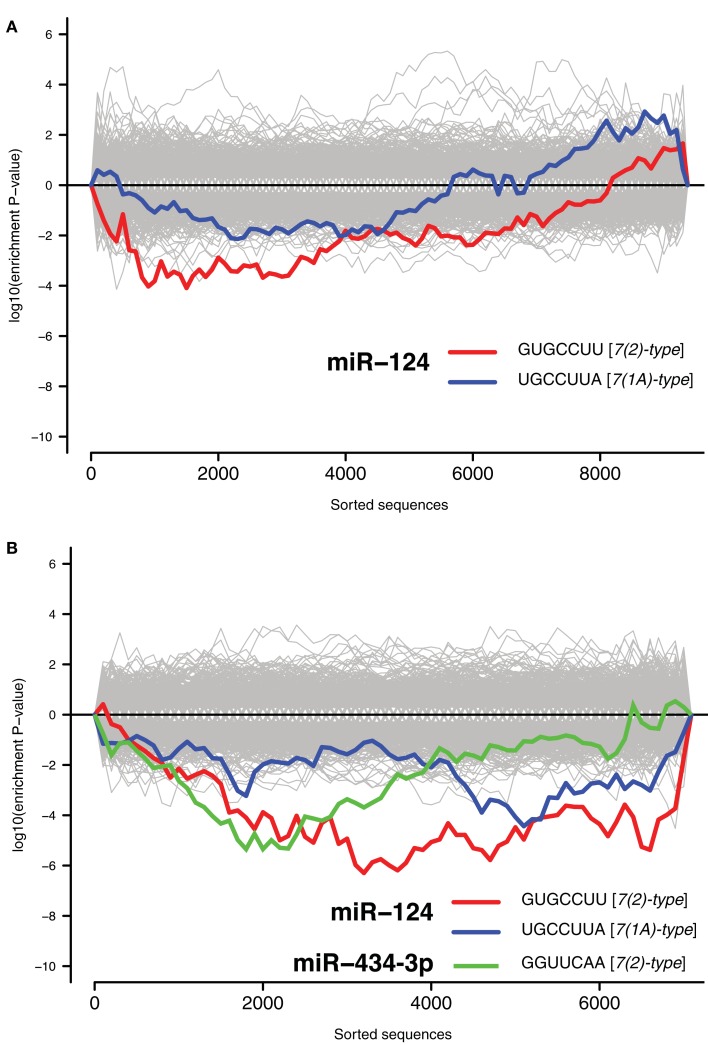
**Signal for activity of endogenous miR-124 and miR-434-3p**. The *x*-axes represent 3′UTRs corresponding to expressed genes sorted from most downregulated to most upregulated in comparison of **(A)** cultures treated with KCl and untreated cultures and **(B)** mouse hippocampi treated with kainate and untreated hippocampi (Akahoshi et al., 2007). *P*-values are calculated using the Sylamer method (van Dongen et al., 2008). Positive values on the *y*-axes represent nucleotide word enrichment [+|log_10_(*P*-value)|] and negative values represent depletion [−|log_10_(*P*-value)|]. The red and blue lines show enrichment profiles of seed matching sites for miR-124 [7(2)-and 7(1A)-types; Bartel, 2009], the green line for miR-434-3p [7(2)-type], and the gray lines – seed matching sites for other miRNAs.

Recent reports have identified a role for miRNA in regulation of mRNA during stress responses (Leung and Sharp, 2010). We therefore examined the potential role of miRNAs in regulating NCR mRNAs. It has previously been demonstrated repeatedly that miRNAs induce coordinated changes in expression of their targets (Farh et al., 2005; Lim et al., 2005). Therefore, we employed Sylamer, a statistical analysis of all known miRNA target sites in 3′UTRs (van Dongen et al., 2008). Sylamer looks for biases in the distribution of target sites in lists of 3′UTRs that are ordered according to the magnitude of differential expression of the corresponding transcripts. If a particular miRNA is overexpressed and has a direct impact on differential expression, then 3′UTRs of downregulated transcripts will be enriched in target sites of that miRNA, but not other miRNAs. On the other hand, if a miRNA is non-functional, then the 3′UTRs of upregulated transcripts will be enriched in target sites of that miRNA. With Sylamer we identified seed matching sites of miR-124 as the most enriched in mRNAs upregulated during the transcriptome response, and thus implicated miR-124 as a potential regulator of the NCR mRNAs (Figure 1B and Materials and Methods). miR-124 is a neuron specific miRNA known to play a role in the maintenance of neuronal identity (Conaco et al., 2006). A similar distribution of miR-124 sites was observed in the mRNAs regulated by KCl and kainate (Figure 2). In addition to miR-124, in the data from the brains of mice treated with kainate we detected a bias in distribution of sites for miR-434-3p, a miRNA that is upregulated in development of primary neurons (Manakov et al., 2009). These observations suggest the possibility that endogenous miR-124 and miR-434-3p regulate NCR mRNAs.

### miRNA-mediated inhibition of the response genes

It has been previously demonstrated that overexpression of miRNAs reduces expression of a significant fraction of their targets (Lim et al., 2005; Giraldez et al., 2006; Selbach et al., 2008), while inhibition (knock down) of miRNAs results in elevated abundance of their targets (Conaco et al., 2006; Giraldez et al., 2006; Selbach et al., 2008). Therefore, comparison of mRNA transcriptome data following treatment with both mimics and inhibitors of miRNAs provides confidence in the targets (Lim et al., 2005; Giraldez et al., 2006). Indeed, we found that contrast of expression between cultures transfected with mimics (i.e., miRNA overexpression) and those transfected with inhibitors (i.e., miRNA knock down) results in bigger and more significant differential expression than contrast of overexpression and simple mock transfection (results not shown). In addition to serving as an efficient contrast for differential expression analysis, the use of miRNA inhibitors provides additional confidence that identified changes in gene expression are directly related to the endogenous function of the inhibited miRNA (see Discussion). Therefore, to identify miRNA targets, we transfected primary neurons with mimics and inhibitors of miR-124 and miR-434-3p or controls including miRNAs that are downregulated in mature neuronal cultures (miR-143, miR-145, miR-25; Manakov et al., 2009). In addition, we transfected cultures with the mimic of *Caenorhabditis elegans* miRNA (cel-miR-67) and compared it against mock transfection (inhibition is not possible for this miRNA, as it is not endogenous to mouse primary neurons).

**Figure 3:**
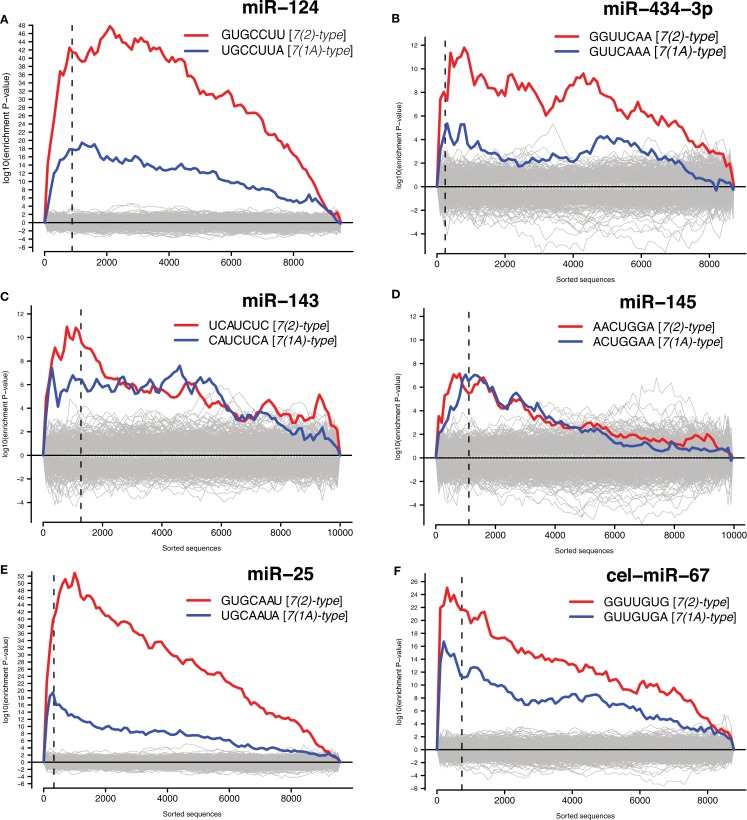
**Identified putative direct miRNA targets**. The *x*-axes represent 3′UTRs corresponding to genes sorted from most downregulated to most upregulated in comparison to cultures transfected with **(A)** miR-124 mimic and inhibitor, **(B)** miR-434-3p mimic and inhibitor, **(C)** miR-143 mimic and inhibitor, **(D)** miR-145 mimic and inhibitor, **(E)** miR-25 mimic and inhibitor, **(F)** ce-miR-67 mimic and mock transfected cultures. Sequences to the left of the vertical dashed lines correspond to genes downregulated with differential expression adjusted *P* < 0.05. Putative targets of miRNAs are defined as downregulated genes (adjusted *P* < 0.05) encoding transcripts with one or *7(2) or 7(1A)-type seed matching sites* (Bartel, 2009) in 3′UTRs. Section “Materials and Methods” for details.

We have then conducted the statistical analysis of miRNA binding site distribution in 3′UTRs of all transcripts detected by microarrays, and found that downregulated transcripts were enriched in the sites for the transfected miRNAs (Figure 3 and Materials and Methods). Seed matching site containing transcripts, that are downregulated in analogous experiments, were shown to be enriched in validated miRNA targets (Lim et al., 2005; Giraldez et al., 2006; Selbach et al., 2008). Therefore we compiled lists of dozens to hundreds of putative direct targets for the six transfected miRNAs (Data Sheet 1 in Supplementary Material).

**Figure 4:**
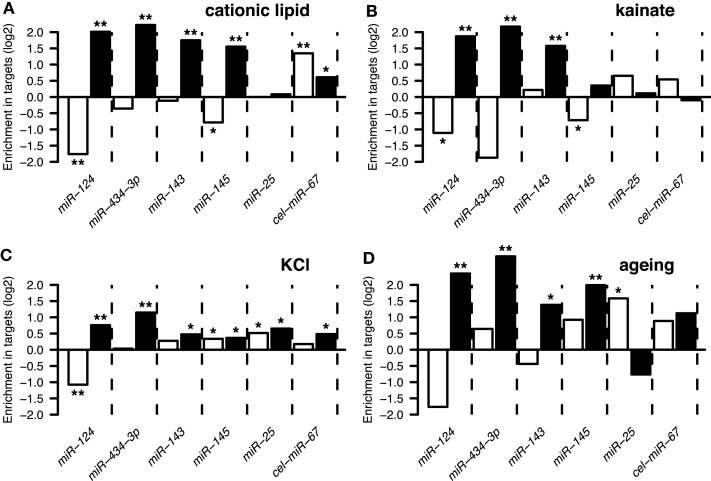
**The *y*-axis show enrichment of miRNA targets in genes that were downregulated (white bars) or induced (black bars) by four types of stressful treatments of neurons and the brain (Data Sheet 2 in Supplementary Material)**. These treatments are described in Results Sections “Induction of Stress Response Genes by the Transfection reagent” and “Other Treatments Also Induce the Response”: **(A)** treatment of primary neuronal cultures with cationic lipid transfection reagent; **(B)** treatment of the brain with kainate; **(C)** treatment of primary neuronal cultures with KCl; **(D)** ageing of the human brain. A single asterisk indicates hypergeometric *P* < 0.05, double indicates *P* < 0.001. The set of 10,849 genes detected by Illumina microarrays (see Materials and Methods) in mock transfected and untransfected cultures was used as the universe for the hypergeometric tests.

**Figure 5:**
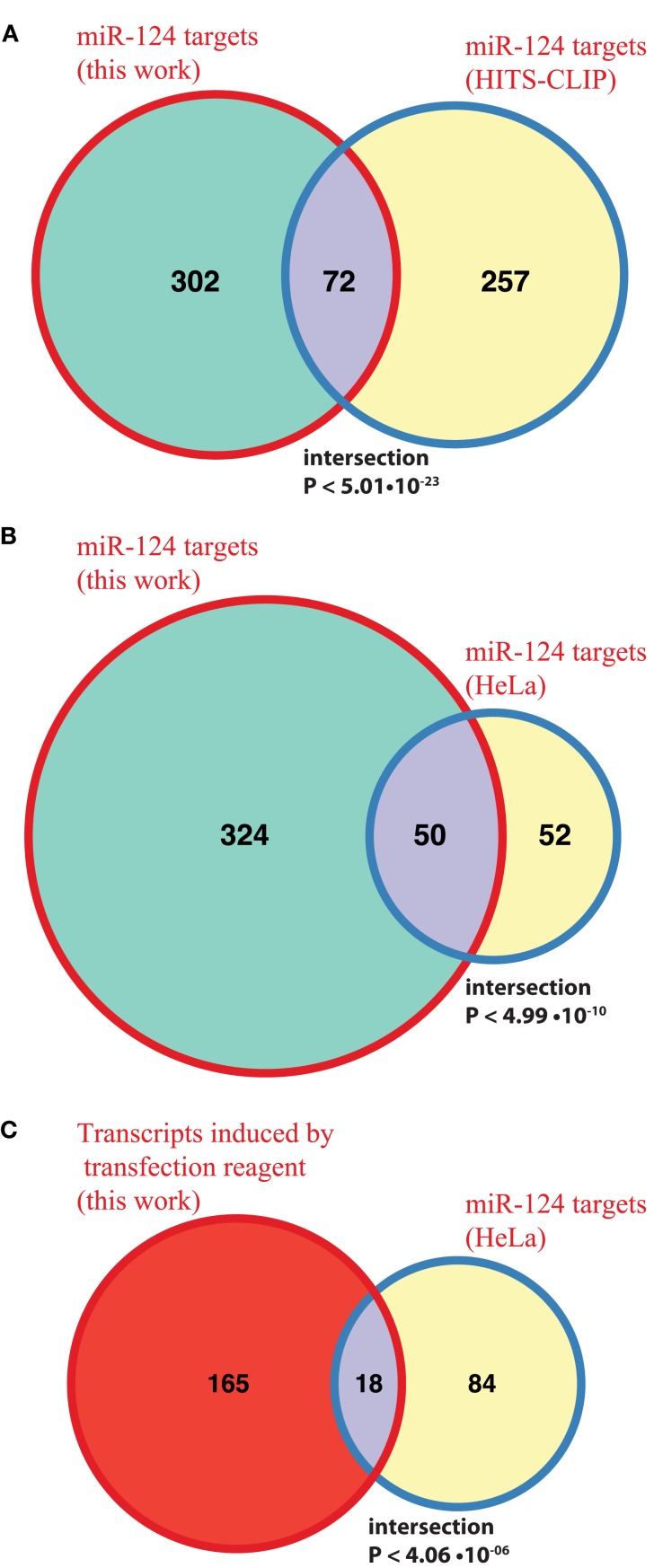
**Comparison with previously published miR-124 targets**. **(A,B)** The Venn diagrams show counts of putative direct targets of miR-124 that were inferred from the transfection experiments in this work and from the published HITS-CLIP sequencing (Chi et al., 2009) and microarray (Lim et al., 2005) experiments. The latter study is based on HeLa transfrection experiment. The mouse homologs of human miR-124 targets were retrieved from HomoloGene http://www.ncbi.nlm.nih.gov/homologene; **(C)** the Venn diagram shows counts of miR-124 targets reported in HeLa study (Lim et al., 2005) and transcripts induced by transfection alone in our experiment. In all cases the test universe was 3,465 mouse transcripts, with 3′UTRs containing one or more 7(2) or 7(1A)-type seed matching site for miR-124. The test universe was a complete list of mouse genes encoding transcripts with 3′UTRs containing one or more 7(2) or 7(1A)-type seed matching site for miR-124, and which were represented on Illumina Sentrix BeadChip Array Mouse-WG6 v2 microarray platform (3,465 genes in total). The text shows fold enrichment above what is expected by chance alone and the hypergeometric *P*-value for the intersection.

Following derivation of putative miRNA targets, we assessed whether the identified targets were enriched among NCR mRNAs, therefore implicating miRNAs in regulation of the NCR. Indeed, analysis of the mRNA targets of miR-124 and miR-434-3p showed that they were significantly enriched in all four NCR sets (transfection, KCl, kainate, aging; Figure 4). Targets of the four transfected non-neuronal miRNAs were also enriched in some of the sets, although these effects were smaller and less consistent than for the two neuronal miRNAs (Figure 4). The targets that we identified for miR-124 were consistent with previously published lists of miR-124 targets (Lim et al., 2005; Chi et al., 2009; Figures 5A,B). These results confirm that increasing or decreasing the levels of miR-124 and miR-434-3p reduces or increases NCR mRNAs respectively.

## Discussion

We found that challenging neurons with different stimuli induces a transcriptome response involving a large set of mRNAs, observed *in vitro* and *in vivo*. This response changes the expression of a set of stress-related pathways and is triggered by a wide variety of exogenous challenges including treatment of primary neurons with a blank cationic lipid transfection reagent, neuronal activation, and aging. A significant proportion of the transcripts activated by the transfection reagent were also activated by other stresses (Figure 1A). Due to this similarity, we refer to changes in the transcriptome during stresses as a NCR. We observed enrichment of miR-124 and miR-434-3p seed matching sites in 3′UTRs of transcripts that were induced during NCR (Figures 1B and 2). Next, we tested whether the induced transcripts were enriched in direct targets of these miRNAs. Purely computational miRNA target prediction algorithms, such as TargetScan (Friedman et al., 2009), PicTar (Krek et al., 2005), or miRanda (Griffiths-Jones et al., 2008), are prone to false positive predictions. Therefore we experimentally derived targets of a selection of miRNAs using an approach that was previously published and validated (Lim et al., 2005; Giraldez et al., 2006). We defined targets as transcripts that both have one or more seed matching sites for a miRNA and, at the same time, are significantly inhibited during overexpression of that miRNA (Materials and Methods). Indeed, we found that NCR mRNAs are preferred targets of two neuronal miRNAs, miR-124 and miR-434-3p (Figure 4). Therefore, these miRNAs can act to modulate or buffer the response at the whole transcriptome level by destabilization of hundreds of transcripts that are induced during the response. The effect of stress on transcriptome can be viewed as a deviation from homeostatic equilibrium that is associated with the unperturbed differentiated state. Therefore, the capacity of miRNAs to inhibit hundreds of transcripts that are activated by stress makes them candidate stabilizers of the normal, homeostatic state of the transcriptome.

To our knowledge, our report is the first to connect a broad targeting capacity of miRNAs and transcriptome wide changes in gene expression that are observed in stresses. We hope that our study will encourage future mechanistic investigation of the principles that govern this relationship. For example, it is possible that miRNAs themselves are inhibited during stress, and that this is the mechanism for the concomitant relief of miRNA-mediated regulation. It is also possible that upon stress, in a fashion similar to protein components of miRNA effector complex (Leung et al., 2006), miRNAs themselves may relocate to cellular compartments where they are kept separate from their targets, enabling induction of normally repressed stress pathways. We believe that addressing these questions will provide an important contribution to understanding of the molecular mechanisms and cellular functions of miRNAs.

Interestingly, one of the treatments that triggered NCR was the transfection procedure itself (i.e., treatment of neurons with a blank cationic lipid transfection reagent). We believe this is the first report of activation of miRNA targets linked to the use of a common cationic lipid transfection reagent. The observed induction of putative direct targets of miR-124, as well as of other miRNAs, raises an important warning sign for miRNA research, because stress caused by experimental procedures, such as transfection, inadvertently induces expression of miRNA targets. Subsequently, inhibition of these targets is observed when miRNA mimic is added to the cells. Therefore, it is important to realize that miRNA targets observed in transfection experiments should be understood in the context of cellular stress associated with the transfection.

We identified enrichment of miRNA targets among genes that respond to stresses in primary neurons (NCR genes). It remains to be established whether NCR is specific to neurons, or if it is related to stress response programs in other cell types. In fact, we propose that miRNA targets are enriched in stress response genes not only in neurons. To look for support of this proposition we examined targets of a neuronal miRNA that were also identified in a transfection experiment, but in a non-neuronal system. HeLa cells were transfected with miR-124 mimic (Lim et al., 2005). Of the miR-124 targets reported in that study, 102 had one-to-one homology to mouse genes and were detected by microarrays in our experiments (Materials and Methods). Of these, 18 genes (17.6%, ∼3.3 times more than expected by chance, *P* < 4.06 × 10^−06^) were activated during mock transfection of primary neurons in our study (Figure 5C). This relationship between miR-124 targets in HeLa and genes activated by mock transfection of primary neurons is significant, but it is weaker than that observed by us in primary neurons (Figure 4A). The latter may be due to the fact that a set of HeLa genes activated during transfection is substantially different from the NCR set. Unfortunately, to our knowledge, the stress response associated with the use of transfection reagent has not been directly studied, even in a popular system like HeLa cells. If genes activated by transfection stress in non-neuronal cell types are reported, it will be interesting to see whether enrichment of targets of a miRNA in these cells, especially of miRNAs that are specific to the cell type in question, will be at the same level as observed in our study.

Recent developments in high throughput proteomics allow quantitative profiling of the global dynamics in abundances of thousands of proteins (Schwanhausser et al., 2011; Geiger et al., 2012). It is likely that we have already identified a near-complete set of stress activated miR-124 and miR-434-3p targets, as it was shown for mammalian miRNAs to primarily act by regulating mRNA abundance (Guo et al., 2010). However, it remains possible that some targets were missed in our study of the transcriptome. In the future it will be important to profile protein changes that take place in primary neurons upon perturbation of miR-124 and miR-434-3p expression. Also, it is not known how quickly miRNA-mediated changes in the transcriptome translate into changes in the whole proteome, and we encourage investigation of this issue in the context of the NCR and related stress responses, where we found individual single miRNAs to be capable of dampening expression of hundreds of mRNAs.

In the marine invertebrate *Aplysia californica*, miR-124 has been shown to constrain synaptic plasticity (long-term facilitation) induced by neuronal activation (Rajasethupathy et al., 2009). Also, ablation of all miRNAs in the adult mouse brain increased synaptic plasticity (Konopka et al., 2010). We hypothesize that changes in the neuronal transcriptome during learning are akin to those of the NCR in that targets of miRNAs are being activated. In such a scenario, miRNAs provide resilience to the transcriptome against different exogenous and endogenous stimuli, like those inducing plasticity or stress responses. Manipulating miRNAs may not only influence mechanisms of learning and memory (Rajasethupathy et al., 2009; Konopka et al., 2010) but also mechanisms engaged by stress, neurotoxicity, and age-related events.

## Conflict of Interest Statement

The authors declare that the research was conducted in the absence of any commercial or financial relationships that could be construed as a potential conflict of interest.

## Supplementary Material

The Supplementary Material for this article can be found online at http://www.frontiersin.org/Neurogenomics/10.3389/fnins.2012.00156/abstract

Supplementary Data Sheet 1 **Putative targets of transfected miRNA derived from mRNA profiling data**.

Supplementary Data Sheet 2 **Gene Lists**.

## Footnotes

^1^http://www.ebi.ac.uk/arrayexpress/

^2^http://support.illumina.com/array/array_kits/mousewg-6_v2_expression_beadchip_kit/downloads.ilmn
